# Structural basis of multitasking by the apicoplast DNA polymerase from *Plasmodium falciparum*

**DOI:** 10.1093/nar/gkaf1005

**Published:** 2025-10-16

**Authors:** Anamika Kumari, Theodora Enache, Timothy D Craggs, Janice D Pata, Indrajit Lahiri

**Affiliations:** Molecular Microbiology, School of Biosciences, The University of Sheffield, Sheffield S10 2TN, United Kingdom; Molecular Microbiology, School of Biosciences, The University of Sheffield, Sheffield S10 2TN, United Kingdom; School of Mathematical and Physical Sciences, The University of Sheffield, Sheffield S10 2TN, United Kingdom; Nucleic Acids Institute, The University of Sheffield, Sheffield¸ S10 2TN, United Kingdom; Exciting Instruments, Block 5, Pennine Five, 18 Hawley Street, Sheffield S1 4WP, United Kingdom; Wadsworth Center, New York State Department of Health, Albany, NY 12208, United States; Department of Biomedical Sciences, University at Albany, Albany, NY 12208, United States; Molecular Microbiology, School of Biosciences, The University of Sheffield, Sheffield S10 2TN, United Kingdom; Nucleic Acids Institute, The University of Sheffield, Sheffield¸ S10 2TN, United Kingdom; Florey Institute of Infection, The University of Sheffield, Sheffield S10 2TN, United Kingdom

## Abstract

*Plasmodium falciparum* is a eukaryotic pathogen responsible for the majority of malaria-related fatalities. *Plasmodium* belongs to the phylum Apicomplexa and, like most members of this phylum, contains a non-photosynthetic plastid called the apicoplast. The apicoplast has its own genome, replicated by a dedicated replisome. Unlike other cellular replisomes, the apicoplast replisome uses a single DNA polymerase (apPol). This suggests that apPol can multitask and catalyse both replicative and lesion bypass synthesis. Replicative synthesis relies on a restrictive active site for high accuracy while lesion bypass typically requires an open active site. This raises the question: how does apPol combine the structural features of multiple DNA polymerases in a single protein? Using single-particle electron cryomicroscopy (cryoEM), we have solved the structures of apPol bound to its undamaged DNA and nucleotide substrates in five pre-chemistry conformational states. We found that apPol can accommodate a nascent base pair with the fingers in an open configuration, which might facilitate the lesion bypass activity. In the fingers-open state, we identified a nascent base pair checkpoint that preferentially selects Watson–Crick base pairs, an essential requirement for replicative synthesis. Taken together, these structural features might explain how apPol balances replicative and lesion bypass synthesis.

## Graphical Abstract

**Graphical Abstract:**
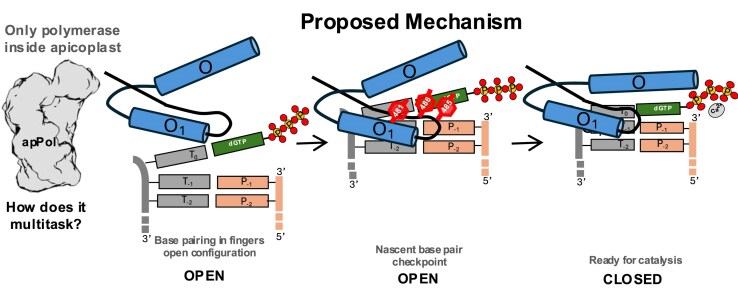

## Introduction

*Plasmodium* is a parasitic protozoan responsible for causing malaria. This parasite belongs to the phylum Apicomplexa and, like most members of this phylum, *Plasmodium* contains an essential, non-photosynthetic plastid called the apicoplast [1]. *Plasmodium* apicoplast has its own circular 35 kb A/T-rich genome coding for a chaperone as well as proteins and RNA involved in apicoplast gene expression [2]. Apicoplast DNA is copied by a dedicated apicoplast replisome. Compared to other cellular replisomes, the apicoplast replisome functions with very few proteins. For instance, all cellular replisomes use replicative polymerases to accurately copy undamaged DNA and specialized translesion synthesis (TLS) and repair polymerases to bypass damaged DNA and perform DNA repair. Apicoplast replication, however, is carried out by a single DNA polymerase called apPol [3–5].

**Figure 1.**
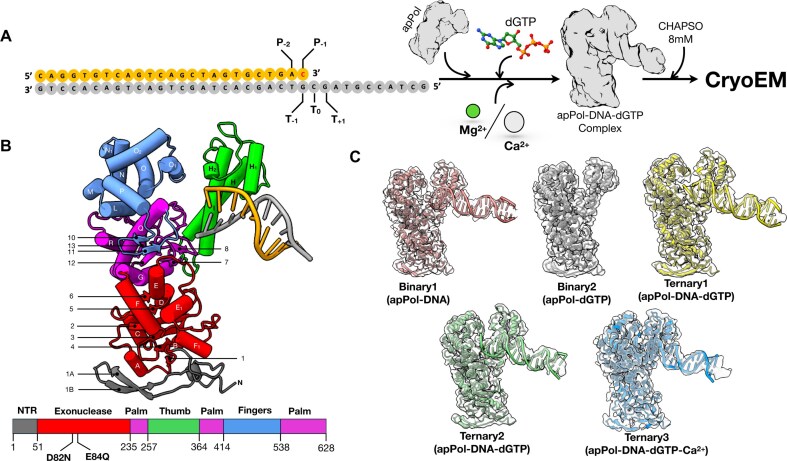
CryoEM of apPol pre-chemistry complexes. (**A**) apPol complex assembly scheme. A synthetic primer/template DNA (primer: orange; template: grey) was incubated with apPol and dGTP in the presence of either Mg^2+^ or Ca^2+^ to form apPol pre-chemistry complex and CHAPSO was added just before cryoEM grid preparation. The templating base (T_0_) and the ones immediately 5′ and 3′ to it (T_+1_ and T_−1_, respectively), along with the two bases at the 3′ end of the primer strand (P_−2_ and P_−1_), are highlighted. The P_−1_ base (dC; red) is dideoxy terminated when Mg^2+^ is used as the divalent metal. (**B**) Atomic model (tube and arrow representation) of the 3.2 Å consensus map from dataset1 along with the domain organization of apPol. The domains are coloured as follows (N-terminal region (NTR): grey; 3′–5′ proofreading exonuclease: red; palm: magenta; thumb: green and fingers: blue). The exonuclease active site residues (D82 and E84) that have been mutated to N and Q, respectively, in all the apPol constructs used in this study are highlighted. The secondary structural elements are labelled based on structure alignment with Klenow fragment (PDB id: 1KLN)^14^. (**C**) Atomic models of the two binary (apPol–DNA (binary1): salmon pink; apPol–dGTP (binary2): grey) and three ternary complexes of apPol–DNA–dGTP (ternary1, ternary2, and ternary3: yellow, green, and blue, respectively) fitted to their respective cryoEM maps (transparent grey).

The structures of most DNA polymerases solved to date resemble a right hand with the enzyme divided into the thumb, palm, and fingers subdomains [6, 7] (Fig. 1). Typically the thumb interacts with the DNA duplex, stabilizing the DNA on the polymerase. The catalytic aspartic acid residues involved in the two-metal-ion mechanism of nucleotide addition [8] are located in the palm domain, while the fingers is involved in ensuring accuracy of the DNA synthesis. The replicative polymerases possess an additional 3′ to 5′ exonuclease activity, which resides in the proofreading exonuclease domain of these enzymes or in a separate subunit of the replisome.

Most DNA polymerases, including apPol, follow the same overall catalytic cycle for DNA synthesis [5, 9–12] (Supplementary Fig. S1A). The pre-chemistry steps involve binding of DNA and nucleotide substrate (Supplementary Fig. S1A, Steps 1 and 2) followed by the transition to a catalytically competent pre-chemistry ternary complex (Supplementary Fig. S1A, Step 3). In most replicative polymerases, this transition involves a large-scale conformational change in the fingers domain termed fingers closure. Fingers closure leads to the formation of a restrictive active site, favouring a Watson-Crick base pairing between the templating base (T_0_) and the incoming dNTP [13, 14]. In TLS polymerases, the fingers closing is typically less pronounced or absent altogether [15–17], leading to a more accessible active site that can accommodate lesions and bypass them. Fingers closing is followed by the chemical step of nucleotide addition (Supplementary Fig. S1A, Step 4) using the two-metal-ion or three-metal-ion mechanism [8, 18, 19]. The post-chemistry steps involve the release of the inorganic pyrophosphate byproduct (PP_i_) followed by translocation along the DNA (Supplementary Fig. S1A, Steps 4 and 5, respectively).

Apicoplast DNA undergoes reactive oxygen species-mediated oxidative damage, and since apPol is the only polymerase in the apicoplast, this enzyme is anticipated to perform both replicative and lesion bypass DNA synthesis [4]. In fact, kinetic analysis shows that apPol can bypass oxidative lesions, including 8-oxo-7,8-dihydro-2′-deoxyguanosine (8-oxodG), 5,6-dihydroxy-5,6-dihydro-2′-deoxythymine (thymine glycol), and 2-oxo-1,2-dihydro-2′-deoxyadenine (2-oxodA), with varying degrees of extensions past the lesions [4, 5].

ApPol belongs to the A-family of DNA polymerases but is only distantly related to the well-studied A-family polymerases, including bacterial DNA polymerase I (Pol I), T7 DNA polymerase, and mitochondrial DNA polymerase gamma [20]. Instead, apPol belongs to a poorly studied clade of A-family DNA polymerases, and members of this clade are found in all domains of life [21]. Recent structures of apo, post-chemistry binary, and pre-chemistry ternary complexes of apPol show how the enzyme interacts with its substrates [22, 23]. However, these structures do not shed light on the conformational changes undergone by apPol as it moves through the different pre-chemistry states and prepares for catalysis.

We have used electron cryomicroscopy (cryoEM) to solve a series of structures of apPol in complex with an undamaged primer/template DNA and the correct incoming dNTP (Fig. 1). These structures elucidate the conformational changes of apPol critical for the pre-chemistry steps. We show that, unlike other A-family polymerases, the pairing between the templating base (T_0_) and incoming nucleotide occurs with the fingers domain of apPol in the open conformation. Moreover, we propose a molecular checkpoint specific to apPol that might allow this polymerase to perform TLS without sacrificing the accuracy needed for replicative synthesis.

## Materials and methods

### DNA substrates

All DNA oligonucleotides were purchased from Integrated DNA Technology (USA). For dataset1, the primer strand was synthesized with a dideoxy-C at the 3′ terminus. Primer/template DNA substrates were generated by annealing the primer and template DNA strands (sequences shown in Fig. 1A and Supplementary Fig. S1B). All annealing reactions were performed using a 1:1.1 ratio of primer and template DNA in annealing buffer [10 mM Tris–Cl (pH 7.5) and 50 mM NaCl]. The sample was heated to 95°C for 5 min, followed by gradual cooling to 25°C.

### Overexpression and purification of apPol

For both the datasets, we have used a construct of apPol with two point mutations (D82N and E84Q) at the active site of the proofreading exonuclease domain (Fig. 1B). This construct has been referred to as apPol throughout the manuscript. For primer extension assays to determine the steric gate residue, an apPol construct with an additional point mutation (E415A or Q548A) was used. All the constructs had an N-terminal hexa-histidine tag followed by a tobacco etch virus protease cleavage site. The constructs were codon optimized for expression in *Escherichia coli* and were synthesized and cloned into the pETDuet1 vector by GenScript Corp. (USA).

All apPol constructs were overexpressed and purified following a previously published protocol [5]. The plasmid was transformed into Rosetta 2(DE3) *E. coli* cells (Merck, USA). The cells were grown in autoinduction terrific broth (Formedium) at 37°C for ∼5 h, followed by a further 20 h of growth at 20°C. The subsequent steps were performed at 4°C. Cell pellets were resuspended in lysis buffer [50 mM Tris–HCl (pH 7.5), 800 mM NaCl, 25 mM imidazole, and 10% glycerol]. To prevent proteolytic degradation of apPol and improve lysis efficiency, ethylenediaminetetraacetic acid (EDTA)-free protease inhibitor tablet (Roche, USA) and lysozyme were added to the resuspended cells. Cells were lysed by sonication and clarified by centrifugation. The clarified lysate was loaded onto a 5 ml HiTrap Chelating HP column (Cytiva, USA) charged with Ni^2+^ and pre-equilibrated with lysis buffer. The unbound protein was washed with 10 column volumes (CVs) of lysis buffer, followed by an additional 10 CV wash with low-salt wash buffer [50 mM Tris–HCl (pH 7.5), 200 mM NaCl, 25 mM imidazole, and 10% glycerol]. ApPol was eluted over a 10 CV linear gradient of imidazole from 25 mM to 1 M. The elution fractions were analysed using sodium dodecyl sulfate–polyacrylamide gel electrophoresis, and fractions containing apPol were pooled and diluted with no-salt buffer [50 mM Tris–HCl (pH 7.5), 25 mM imidazole, and 10% glycerol] to decrease the NaCl concentration to 100 mM. The diluted sample was loaded on a 5 ml HiTrap SP column (Cytiva, USA) pre-equilibrated with Buffer A [50 mM Tris–HCl (pH 7.5), 100 mM NaCl, 5 mM EDTA, 5 mM β-mercaptoethanol, and 10% glycerol]. Unbound protein was washed with 10 CVs of Buffer A, and apPol was eluted with a linear gradient of 0.1 to 1 M NaCl (10 CVs). For further purification, the eluent of the cation exchange column was concentrated and loaded on a Superdex 200 Increase 10/300 size-exclusion chromatography column (Cytiva, USA) pre-equilibrated with storage buffer [50 mM Tris–Cl (pH 7.5), 200 mM NaCl, 2 mM DTT, and 20% glycerol]. Fractions containing pure apPol were pooled, concentrated, flash-frozen in liquid nitrogen, and stored at −80°C. Protein concentration was determined based on the theoretical extinction coefficient of 64180 M^–1^ cm^–1^. The hexa-histidine tag was not removed prior to using the protein.

### Primer extension assays

All primer extension assays were performed at 37°C in reaction buffer containing 20 mM Tris–Cl (pH 7.5), 10 mM MgCl_2_, 30 mM NaCl, 1 mM DTT, 0.1 mg/mL bovine serum albumin, and 5% glycerol. A multiple nucleotide primer extension assay was performed to verify apPol’s activity in the presence of 8 mM CHAPSO. A final concentration of 480 nM apPol was incubated with 200 nM primer/template DNA with the primer strand having a FAM label at the 5′ end (FAM-P/T; Supplementary Fig. S1B). The reaction was initiated by adding 250 μM of each dNTP. The reaction was quenched after 10 or 20 s by adding 250 mM EDTA.

To investigate the role of Y481, Y485, and Y486 in the primer extension activity of apPol, single nucleotide primer extension assays were performed with apPol and apPol^Y481A_Y485A_Y486A^. The assay condition is similar to the one described earlier with the following changes. CHAPSO was not included in the reaction buffer, and instead of multiple nucleotides, only a single nucleotide (either dGTP or dATP) was added.

To ascertain the identity of the steric gate residue of apPol single nucleotide primer extension assays were performed with a final concentration of 1 μM apPol (or apPol^E415A^ or apPol^Q548A^). The enzyme was incubated with 50 nM FAM-P/T and 125 μM of dGTP or GTP was added to initiate the reaction. The reactions were incubated for varying time intervals ranging from 0 to 120 s and then quenched with EDTA.

The products (primer strand extended by multiple or a single nucleotide) were analysed on 15% polyacrylamide (19:1) urea gels run at 40°C–45°C. The gels were imaged on a Typhoon FLA7000 LASER-based scanner (Cytiva, USA) using an excitation wavelength of 488 nm (blue LASER) and an emission cutoff at 525 nm to detect the fluorescence signal from the 5′ FAM label of the primer strand.

For single nucleotide primer extension assays, gel bands were quantitated using ImageQuant software (Cytiva, USA), and the fraction of DNA extended was calculated using the following equation.


\begin{eqnarray*}
Fraction\ of\ DNA\ extended\ = \ \left( {IE - IB} \right) & \div & \ (\left( {IE - IB} \right)\ \nonumber\\ &+& \ \left( {IU - IB} \right)),
\end{eqnarray*}

where *IE* is the intensity of the extended primer band, *IB* is the intensity of the gel background, and *IU* is the intensity of the unextended primer band. The data were plotted using RStudio (Posit). A detailed list of R packages used for graph plotting is provided in Supplementary Table S1.

### CryoEM sample preparation

For dataset1, a ternary complex of apPol, DNA, and dGTP was prepared by incubating 20 μM apPol with 30 μM primer (3′dideoxy-terminated)/template DNA (Fig. 1A) and 6 mM dGTP at 37°C for 10 min in complex-formation buffer [20 mM Tris–Cl (pH 7.5), 10 mM MgCl_2_, 65 mM NaCl and 1 mM DTT]. The ternary complex for dataset2 was formed similarly to dataset1 with the following three alterations. First, the primer strand was not 3′ dideoxy terminated; second, the complex-formation buffer had 10 mM CaCl_2_ instead of 10 mM MgCl_2_; and third, 4 mM of dGTP was added instead of 6 mM.

8 mM CHAPSO was added to the samples immediately before grid preparation to prevent denaturation of the complex by potentially stopping it from going to the air-water interface [24]. CryoEM grids were prepared using a Leica EM GP automatic plunge freezer (Leica Microsystems). Four microlitres of the sample was applied to a glow-discharged Quantifoil R1.2/1.3 400 mesh holey carbon grid (SPT Labtech). Excess liquid was blotted for 2 s and the grids were immediately plunge-frozen in liquid ethane. The vitrified samples were stored in liquid nitrogen until imaged.

### CryoEM imaging and 3D reconstruction

CryoEM grids were screened on an FEI Tecnai Arctica electron microscope (FEI, USA) and the final high-resolution datasets were collected on a Titan Krios (Thermo Fisher Scientific) operated at 300 kV. Dose-fractionated movies were recorded on a K3 direct electron detector (Gatan Inc.) operated in super-resolution mode. Data collection was automated using EPU (Thermo Fisher Scientific) and the images were collected at a nominal magnification of 81 000× such that the object-level pixel size was 1.06 Å/pixel (super-resolution pixel size: 0.53 Å/pixel). The images were recorded as 1 s movies divided into 70 frames. The total dose and fluence were 70 electrons/Å^2^ and 1 electron/Å^2^/frame, respectively, for dataset1, and 60 electrons/Å^2^ and 1.2 electrons/Å^2^/frame, respectively, for dataset2.

All image processing jobs and three-dimensional (3D) reconstructions (Supplementary Fig. S2) were performed using CryoSPARC (version 4.5.1) [25] (Structura Biotechnology Inc.). The individual super-resolution movie frames were binned by 2 and the frames were aligned using alignparts_lmbfgs [26] as implemented within Cryosparc. The contrast transfer function (CTF) of the micrographs was estimated using the patch CTF routine of Cryosparc.

For dataset1, after removing unsuitable micrographs (micrographs with defocus higher than −3.8 μm, 0.5 CTF fit resolution worse than 6 Å, and relative ice thickness higher than 1.1 were rejected), 14 519 micrographs were retained out of 16 197 for further processing. Particles were picked using Topaz [27]. The training set contained ∼1400 manually picked particles from micrographs with varying defocus. At this stage, 1 922 870 particles were selected. These particles were subjected to multiple rounds of 2D classification and 898 336 particles contributing to optimal 2D class averages were retained for further processing. These particles were used for reference-free 3D reconstruction using ab initio reconstruction [25]. One of the classes (354 534 particles) showed clear features consistent with apPol and was taken forward for further processing. These particles were refined using the non-uniform refinement routine [28] resulting in a consensus map with a nominal resolution of 3.2 Å. Multiple rounds of 3D classification (without alignment) were done, including masked classification [29], focussing on the DNA and nucleotide substrates to resolve the heterogeneity in the dataset. This resulted in four different classes defined as binary1, binary2, ternary1, and ternary2. Residual beam-induced motion and defocus estimation errors of the particles contributing to these classes were rectified using the reference-based motion correction and per-particle CTF estimation routines of Cryosparc. This was followed by 3D refinement (using the non-uniform refinement routine of Cryosparc) of each of the 3D classes, resulting in the final maps.

For dataset2, after removing unsuitable micrographs (micrographs with average defocus greater than −3.8 μm, 0.5 CTF fit resolution worse than 5 Å, full-frame motion greater than 27.4 pixels, and relative ice thickness greater than 1.0 were rejected). 11 231 micrographs were retained out of 11 944 for further processing. Particles were picked using Topaz. The training set contained ∼1400 manually picked particles from micrographs with varying defocus. At this stage, 1 993 221 particles were selected. These particles were subjected to multiple rounds of 2D classification and 238 125 particles contributing to optimal 2D class averages were retained for further processing. These particles were used for reference-free 3D reconstruction using ab initio reconstruction and one class resembling apPol (102 768 particles) was taken forward for further processing. Multiple rounds of heterogeneous refinement were performed to further eliminate particles that do not contribute to high-resolution reconstruction, and 92 787 particles were taken forward. The residual beam-induced motion was rectified, and the defocus of these particles was re-estimated using the reference-based motion correction and per-particle CTF estimation routines of Cryosparc, respectively. This was followed by 3D refinement (using non-uniform refinement) leading to a 3.5 Å map of the ternary3 complex.

The resolutions of all cryoEM maps are reported based on the 0.143 cutoff of the gold-standard Fourier shell correlation (FSC) curves [30, 31]. For better map interpretability, the refined maps were sharpened either by applying a negative B factor [32], estimated during non-uniform refinement, or using DeepEMhancer [33]. For DeepEMhancer-based sharpening, the tightTarget model was used.

### Atomic model building and refinement

The published apo apPol structure [22] (PDB ID: 5DKT) was used as the starting model for apPol. The starting model for the primer/template DNA was generated from the structure of *Bacillus stearothermophilus* DNA polymerase 1 (BSt Pol I) (PDB id: 1LV5) [34] fitted into the sharpened cryoEM maps using the ‘Fit in Map’ tool in ChimeraX [35]. The fitted models were examined in Coot [36], and fits of side-chain rotamers and main chain were optimized either by real-space refinement within Coot or in Isolde [37] and subjected to real-space refinement within Phenix [38]. Ligand restraint for dGTP was generated using eLBOW ligand restraint generation [39] in Phenix and was used in all dGTP refinement. The refined structures were analysed in Coot and rebuilt where necessary to optimize model geometry (assessed by MolProbity [40]) and the fit of the model within the cryoEM map (Supplementary Table S2). To estimate overfitting, FSC curves were calculated between the cryoEM map and the atomic model (map-to-model FSC [32]) in Phenix.

### Structure and sequence analysis

All maps and models were visualized in UCSF ChimeraX and unless otherwise stated, the map density depicted in the figures corresponds to the maps sharpened using DeepEMhancer. Map segmentation was performed using Seggar [41]. For comparison between different apPol complexes, residues 405–411 and 572–585 of apPol were used for alignment. The movies depicting morphs between the different states of apPol were prepared in PyMOL (Schrodinger). Protein sequence alignment was performed using Clustal Omega [42] and the alignment was visualized using Jalview [43].

## Results

We assembled two pre-chemistry ternary complexes of apPol (with an inactive exonuclease domain) bound to a primer/template DNA and the corresponding incoming nucleotide (dGTP) (Fig. 1A and B). To prevent the chemical step of bond formation (Supplementary Fig. S1A, Step 3), we either used a 3′ dideoxy-terminated primer strand with Mg^2+^ as the divalent cation (dataset1) or a non-dideoxy-terminated primer strand with Ca^2+^ as the cation (dataset2). In both datasets 8 mM CHAPSO was used to achieve a uniform particle distribution over a holey carbon grid (Fig. 1A). We performed a multiple nucleotide incorporation assay to ascertain that apPol retained its activity in the presence of CHAPSO (Supplementary Fig. S1B).

We solved the structures of these complexes using cryoEM. For dataset1 the consensus structure was determined to an overall resolution of 3.2 Å (Supplementary Fig. S2). The apPol density was well-resolved, and we could trace the entire polypeptide backbone (Fig. 1B) and identify densities for several amino acid residues. Overall, the apPol structure was similar to the previously reported structure of the apo enzyme [22]. The DNA density was fragmented and there was no clear density for the incoming nucleotide (Fig. 1B and Supplementary Fig. S2). We further classified the particles contributing to the consensus map and could resolve them into four structures (global resolution ranging from 3.8 Å to 4.1 Å) corresponding to apPol–DNA, apPol–dGTP, and two conformational states of apPol–DNA–dGTP complexes (Fig. 1C and Supplementary Fig. S2). From dataset2 we solved an apPol-DNA-dGTP-Ca^2+^ ternary complex structure to a global resolution of 3.5 Å (Fig. 1C and Supplementary Fig. S2). Here both the DNA and nucleotide substrates were clearly visible. By combining all these structures, we describe the events leading up to the chemical step of bond formation by apPol.

### ApPol binds DNA and nucleotide independent of each other

**Figure 2.**
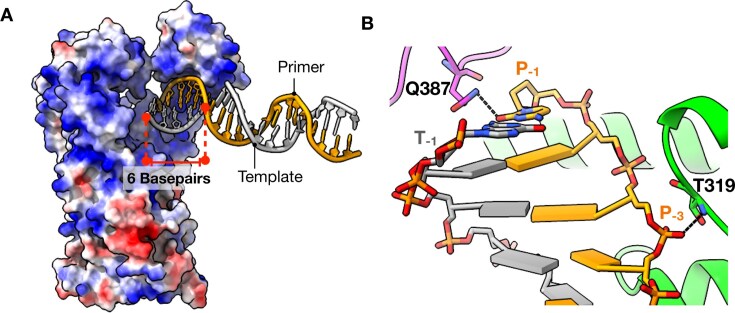
Binary1 complex of apPol. (**A**) Six base pairs (stick representation; red dashed lines) of DNA interact with a positively charged groove (blue) of apPol (surface representation; coloured according to coulombic potential; red: negative charge to blue: positive charge). (**B**) Specific interactions of apPol with the DNA duplex. Colour coding is the same as in Fig. 1B. Potential hydrogen bonds are shown as dashed lines.

The 3D classification of the particles contributing to the consensus structure of dataset1 resulted in two binary complexes: one had apPol bound to DNA (binary1) and the other had apPol bound to the incoming dGTP (binary2) (Supplementary Fig. S2 and Fig. 1C). In binary1 we could detect density for 20 out of the 25 base pairs of the DNA duplex (Fig. 1C and Supplementary Fig. S3A). Of the 20 base pairs, only six were contacted by apPol (Fig. 2A). In the sharpened map no clear density was visible for the template overhang, including the T_0_ base. However, in the unsharpened map we could detect the density for the T_0_ base (Supplementary Fig. S3A), and that guided the register of the DNA duplex. Similar to other A family polymerases, the DNA is mainly contacted by the thumb domain of apPol. Helices H, H_1_, and H_2_ form a positively charged groove that accommodates the minor groove phosphate backbone of the DNA duplex (Fig. 2A). However, consistent with a recent report [23], unlike other A family polymerases, the interactions between the DNA and the thumb are limited in apPol, with a total buried surface area of only 607 Å^2^. We detected two specific contacts: Q387 forms a hydrogen bond with the 3′ base (P_−1_) of the primer strand potentially determining the position of the DNA in binary1, and the backbone nitrogen of T319 interacts with the phosphate backbone of P_−3_ on the primer strand (Fig. 2B). The template strand does not make any significant contact with apPol with a buried surface area of only 174 Å^2^. We note that at the resolution range of the structures reported here (3.2 Å to 4.1 Å), it is not possible to reliably detect density for ordered water molecules, and thus any potential water-mediated interactions have not been analysed throughout this work.

The binary2 structure is similar to that of binary1 except that there is no clear DNA density (Fig. 1C and Supplementary Fig. S2). Instead, we can detect the density for the incoming dGTP near the O helix of the fingers domain (Supplementary Fig. S3C). The base moiety is stabilized by H-bond to Y486 of the O_1_ helix, while the triphosphate forms hydrogen bonds with R390.

**Figure 3.**
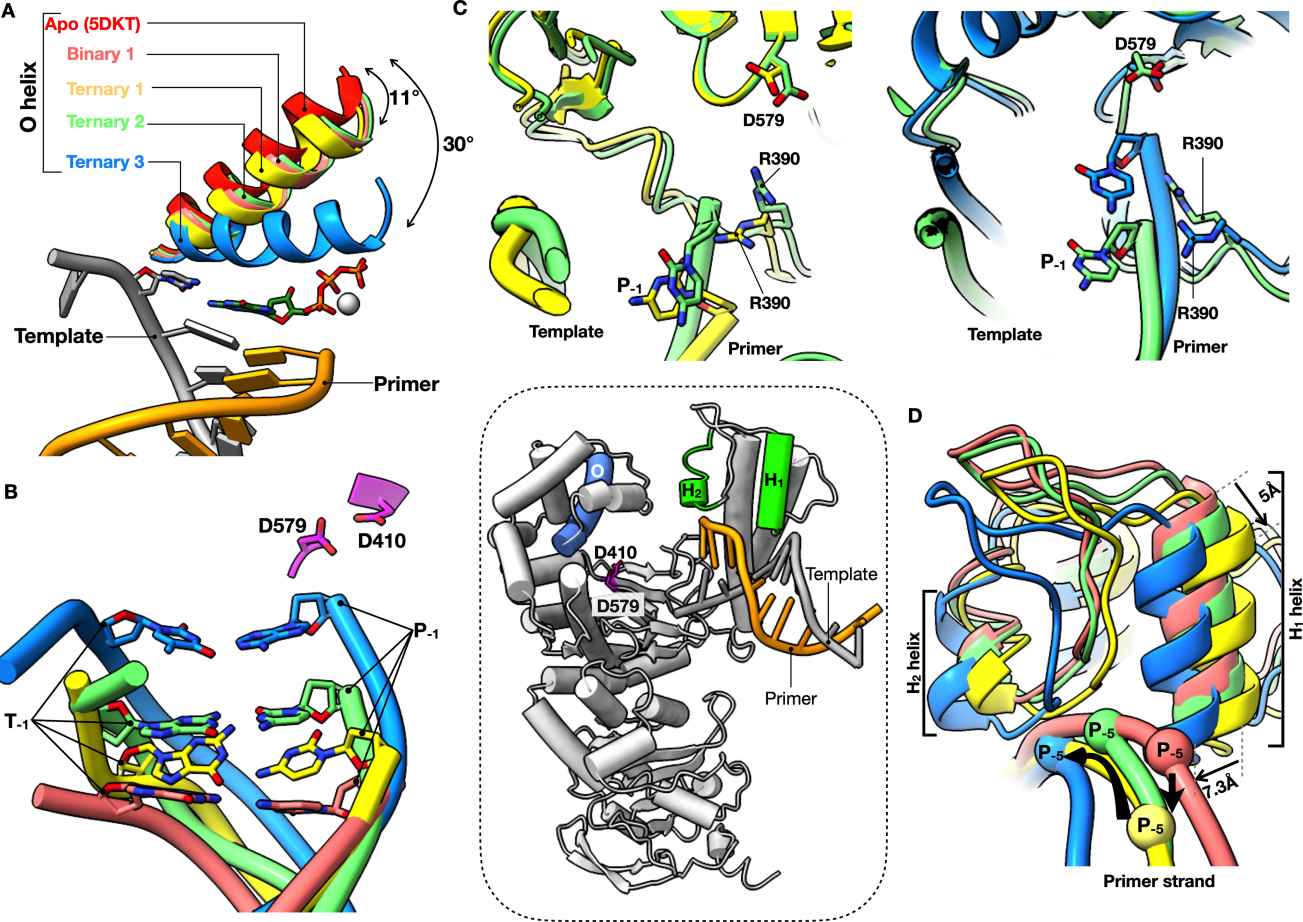
Protein conformation and DNA position changes leading to a catalytically poised apPol complex. (**A**) Position of the O helix (highlighted in blue in the inset) of apPol pre-chemistry complexes with respect to the O helix position of *apo* apPol (PDB id: 5DKT). The primer (orange)/template (grey) DNA, incoming dGTP (green), and Ca^2+^ (white) occupying the position of metal B in ternary3 are shown for reference. The T_0_ base is depicted in stick representation, while all other bases of the DNA are shown as slabs. For angle measurement the C_α_ atom of Y471 of *apo* apPol was selected as the vertex. (**B**) Position of the T_−1_-P_−1_ base pair of binary1, ternary1, ternary2, and ternary3 with respect to the catalytic aspartates (D579 and D410; magenta; position of the aspartates highlighted in the inset). The DNA backbones are shown as ribbons with the T_−1_ and P_−1_ bases shown in stick representation. Only the catalytic aspartases of ternary3 are shown for clarity. We note that the peptide backbones of D579 and D410 of all the apPol complexes were aligned to each other. (**C**) Position of R390 in ternary1 and ternary2 (left) and ternary2 and ternary3 (right) with respect to the 3′ end of the primer. R390, D579, and the P_−1_ nucleotide are shown in stick representation. (**D**) Alterations in thumb and DNA positions among the different apPol complexes. For clarity, only H_1_ and H_2_ helices (highlighted in green in the inset) of the thumb, along with the connecting loop and the DNA primer strands, are shown. The spheres on the primer strands denote the P_−5_ positions and are used to illustrate the DNA motion. Black arrows show the potential thumb (thin arrows) and DNA (thick arrows) motions. Colour coding for all panels: salmon pink: binary1; yellow: ternary1; green: ternary2; blue: ternary3; red: apo. Inset: apPol model from Fig. 1B with the DNA omitted. The protein is coloured white with the O (blue), H_1_ and H_2_ (green), helices and D410 and 579 (magenta) highlighted.

For most DNA polymerases, a transition from the apo to the catalytically poised pre-chemistry ternary complex is accompanied by the closure of the fingers domain [44–48], restricting the active site by forming a lid over the nascent base pair. For A-family polymerases, fingers closure is achieved by a large-scale rotation of the O and N helices towards the nascent base pair [45, 48, 49]. In both binary1 and 2, the O helix is in a predominantly open position with the N-terminal tip of the O helix rotating towards the active site by only ∼11° (Fig. 3A). We will denote this state of the O-helix as quasi-open. Taken together, binary1 and 2 complexes indicate that apPol can engage with the DNA and nucleotide substrates independent of each other.

### ApPol–DNA binary complex base pairs with the incoming nucleotide with the fingers open

We solved the structure of apPol-DNA-dGTP ternary complex in three conformational states (ternary1, 2, and 3) (Fig. 1C). Ternary1 and 2 were resolved through 3D classification of dataset1 while ternary 3 is from dataset2 (Supplementary Fig. S2). The overall protein structure is similar among these states (average C_α_ RMSD between ternary1 and 2 is 1.5 Å, and between ternary1 and 3 and between ternary2 and 3 are 2.8 Å and 2.9 Å, respectively). In ternary1 and 2 the O helix is quasi-open, while in ternary3 the fingers are in a closed conformation with the O helix rotating towards the active site by 30° (Fig. 3A; Movie 1). By comparing the DNA positions and active site configurations of the ternary complexes with those of binary1 (Fig. 3B and C), we could assign the complexes on a reaction pathway starting with the initial interaction of dGTP with the apPol-DNA binary complex to the catalytically poised pre-chemistry state.

**Figure 4.**
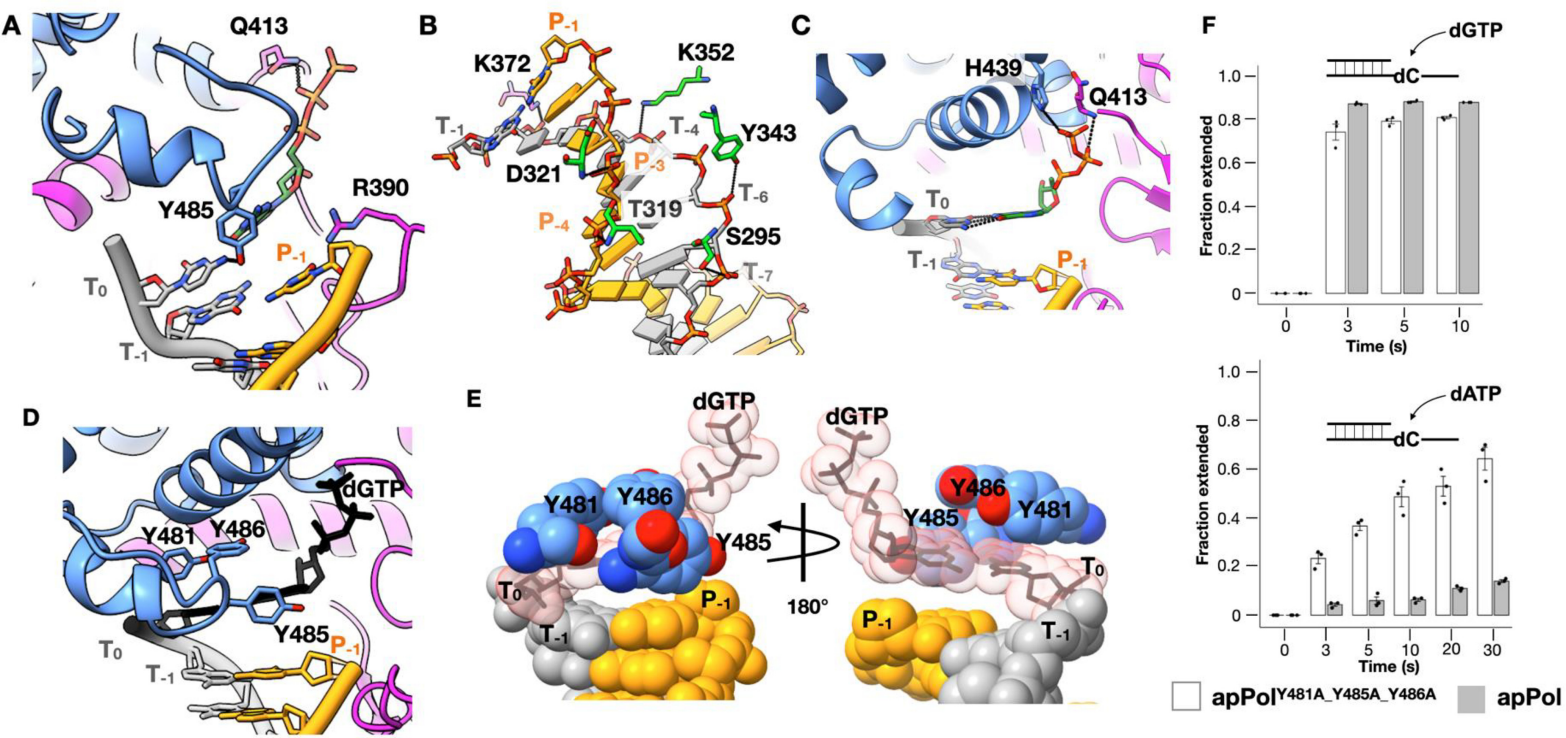
Open ternary complexes of apPol. (**A**) The incoming dGTP in ternary1. Residues interacting with the dGTP and R390 are shown in stick representation. (**B**) Specific interactions of apPol with the DNA duplex in ternary2. The bases are shown as slabs except for T_−1_ and P_−1_, which, along with the interacting residues are shown in stick representation. (**C**) The nascent base pair in ternary2. Residues interacting with the dGTP are shown in stick representation. (**D, E**) Pre-insertion checkpoint formed by Y481, Y485, and Y486 of the O_1_ helix in ternary2. (D) Cartoon representation of the pre-insertion checkpoint with the tyrosines shown in stick form. (E) The three tyrosines and the DNA duplex are shown as spheres with the incoming dGTP and the T_0_ base shown both in sphere and stick representations. Colour coding for all panels is the same as Fig. 1B with the incoming dGTP shown in dark green in panels (A) and (C). The sphere representations of the T_0_ base and the incoming dGTP in panel (E) are shown as transparent salmon pink and the corresponding stick representation panels (D) and (E) are in black. (**F**) Single nucleotide addition by apPol^Y481A_Y485A_Y486A^ (open bar) or apPol (filled bar) incorporating dGTP (correct addition; top panel) or dATP (incorrect addition; bottom panel) opposite dC. 480 nM enzyme was incubated with 200 nM FAM-P/T. 250 µM dGTP or dATP was added to initiate the reaction. The reactions were quenched after various time intervals with 250 mM EDTA and the fraction of starting primer extended by one nucleotide has been plotted as bars. The experiments were performed in triplicates and the individual replicates are shown as black circles with the average value shown as bars. Error bars show the standard deviation.

The ternary1 DNA position is closest to that of binary1 (Fig. 3B), and we assign it as the initial collision ternary complex, capturing an initial contact of the incoming dGTP with the apPol–DNA pre-chemistry binary complex. Going from binary1 to ternary1 the H_1_ and H_2_ helices of the thumb push down on the DNA such that the DNA pivots around P_−3_, and the T_−1_-P_−1_ base pair swings deeper into the palm domain, getting closer to the catalytic aspartates (D410 and D579) (Fig. 3D and B; Movies 2 and 3). In this state the 3′ end of the primer strand is ∼17 Å away from D579 (Supplementary Fig. S4). We could detect 20 base pairs in the duplex DNA but no density was present for the template overhang except for the T_0_ base (Fig. 1C). We could detect the density for the incoming dGTP in between the fingers and palm domains (Supplementary Fig. S3D). The triphosphate moiety of the dGTP is in an extended configuration and is stabilized by Q413 of the fingers domain (Fig. 4A and Supplementary Fig. S3D). Compared to the triphosphate, the densities for the ribose and base were weak, and we could only tentatively model their orientations. We postulate that in ternary1 the incoming dNTP can sample multiple conformations, and once favourable pairing occurs with the T_0_ base, the complex moves to ternary2. In addition to the apPol-DNA contacts of binary1, we note that Y485 of the O_1_ helix of the fingers domain and the T_0_ base are within hydrogen bonding distance (Fig. 4A). Interestingly, the sidechain of R390 is sandwiched between the 3′ end of the primer and the incoming dGTP and acts as a roadblock for the DNA moving towards the fingers (Fig. 4A and Supplementary Fig. S4).

### ApPol-specific O_1_ helix extension acts as a nascent base pair checkpoint

Going from ternary1 to ternary2, R390 adopts an altered conformation, no longer restricting the 3′ end of the primer terminus (Fig. 3C left panel and Supplementary Fig. S4). This altered R390, combined with a rigid body motion of the thumb towards the fingers domain (Fig. 3D), guides the DNA towards the fingers and moves the 3′ end of the primer closer to the catalytic aspartates by ∼6 Å (Fig. 3B and C and Supplementary Fig. S4). In ternary2, the template strand forms multiple hydrogen bonds with the thumb and palm domain residues (Fig. 4B). The incoming dGTP pairs with the T_0_ dC and the pairing is consistent with a Watson–Crick base pair (Fig. 4C). Y481, Y485, and Y486 of the O_1_ helix form a snug cavity for the nascent base pair (Fig. 4D and E and Supplementary Fig. S3E; Movie 4). Mutating these tyrosines to alanines made the mutant apPol (apPol^Y481A_Y485A_Y486A^) error prone. In addition to the correct incorporation of a dGTP, apPol^Y481A_Y485A_Y486A^ could also misincorporate dATP opposite a templating dC (Fig. 4F). Compared to Pol I, the O_1_ helix of apPol is longer by ∼1 turn at the C-terminal end (Supplementary Fig. S5), and Y481, Y485, and Y486, conserved within the apPol clade of A-family polymerases [20], are positioned in this additional helical turn. Unlike the incoming nucleotides of the catalytically poised closed ternary complexes of DNA polymerases, the triphosphate moiety of the incoming dGTP in ternary2 adopts a different orientation with the oxygens of the gamma phosphate curled away from the catalytic aspartates (Fig. 4C and Supplementary Fig. S3E). The dGTP is stabilized by H439 and the backbone of Q413. We could not detect any metal ion density with the incoming dGTP in ternary1 or 2.

### Catalytically poised apPol has the fingers closed around the nascent base pair

**Figure 5.**
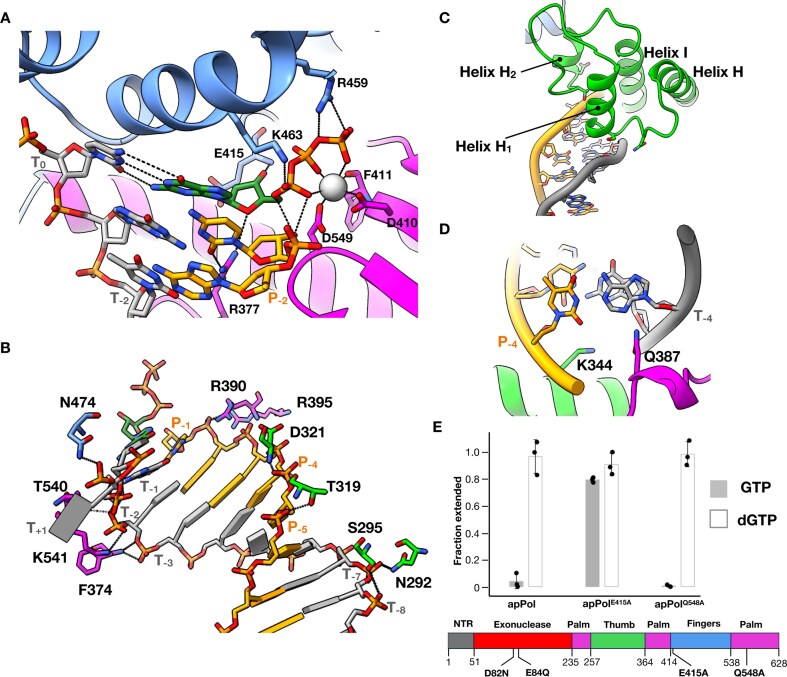
Catalytically poised apPol. (**A**) Polymerization active site of ternary3. Residues interacting with the incoming dGTP are shown in stick representation. (**B**) Specific interactions of apPol with the DNA duplex in ternary3. The bases are shown as slabs except for T_−1_ and P_−1_. These bases, incoming dGTP, and the interacting residues are shown in stick representation. (**C**) Thumb elements of ternary3 interacting with the minor groove backbone of the primer/template DNA. The residues involved in specific contacts are shown in stick representation. (**D**) Top-down view of the primer/template DNA duplex highlighting Q387 and K344 sensing the minor groove geometry of the T_−4_-P_−4_ base pair. Colour coding in panels (A–D) is the same as in Fig. 1B, with the incoming dGTP shown in dark green and Ca^2+^ is represented as white sphere. (**E**) Single nucleotide primer extension assay to validate the steric gate residue. 1 µM apPol (either apPol (Fig. 1B) or apPol with an additional point mutation (E415A or Q548A)) was incubated with 50 nM FAM-P/T. 125 µM GTP (filled bars) or dGTP (open bars) was added to initiate the reaction. The reactions were quenched after 2 minutes with 250 mM EDTA and the fraction of starting primer extended by one nucleotide has been plotted as bars. The experiments were performed in triplicates and the individual replicates are shown as black circles with the average value shown as bars. Error bars show the standard deviation.

To capture ternary3, we used a primer strand with 3′OH but instead of Mg^2+^ we used Ca^2+^ as the divalent metal ion (dataset2) (Fig. 1A). It has been shown that while Ca^2+^ allows the formation of the catalytically poised ternary complex, catalysis by most polymerases is prevented in part due to the subtle differences in coordination geometries and the size difference between the calcium and magnesium ions [19, 50, 51]. Based on the active site arrangement of ternary3 (Fig. 5A), we conclude that this state is the catalytically poised state of apPol. Going from ternary2 to ternary3, the thumb undergoes a rigid body motion towards the fingers, leading to a stronger interaction with the DNA and corkscrew movement of the DNA into the apPol active site (Fig. 3D and B and Supplementary Fig. S4; Movies 2, 3, and 5). The DNA duplex in ternary3 is stabilized through extensive contacts with apPol (Fig. 5B) with a total buried surface area of 1798 Å^2^. In this state the thumb tracks both the backbone geometry and the base pair orientation of the DNA minor groove (Fig. 5C and D; Movie 2), and this might act as a checkpoint for potential misincorporations. In ternary3 the 3′ primer terminal base is stabilized by a minor groove hydrogen bond with R377 (Fig. 5A), placing the P_−1_ C3′ 4.2 Å away from alpha phosphate (P_α_) of the incoming dGTP. Notably, the position of the 3′ end of the primer strand of ternary3 is occupied by R390 in ternary2 (Fig. 3C, right panel). Thus, as the DNA moves into the active site, R390 adopts an altered conformation to accommodate the primer strand (Fig. 3D and Supplementary Fig. S4).

In ternary3 the O helix of the fingers domain clamps down on the nascent base pair, restricting the active site of apPol in preparation for chemistry (Figs 3A and 5A). The closure of the O helix is accompanied by a concomitant movement of the O_1_ helix away from the catalytic aspartates (Movies 1 and 4). The O helix closure pushes the dGTP down into the active site with the triphosphate in a configuration conducive for catalysis (Fig. 5A). The triphosphate is stabilized through interactions with R459 and K463 of the fingers. We could detect the density for metal B, coordinated by the two catalytic aspartates (D579 and 410), F411 backbone, and the triphosphate of the incoming dGTP (Fig. 5A). Interestingly, we could not detect any density for metal A, indicating that metal A might have weaker binding affinity compared to metal B. Instead, we find the 3′OH of the primer strand to form hydrogen bonds with the incoming dGTP. We interpret the ribose pucker of the incoming nucleotide as C3′-endo (Supplementary Fig. S6A), similar to the sugar pucker observed in other catalytically poised DNA polymerases. From the ternary3 structure we identify E415 as the steric gate residue [52]. The C2′ of the dGTP is positioned 3.7 Å above E415 (Fig. 5D). We anticipate that this residue will discriminate against an incoming NTP through steric clash with the 2′OH. In fact, this glutamic acid is conserved in A-family polymerases and acts as the steric gate [14, 52–54]. We performed single nucleotide primer extension assays with both wild type (exonuclease inactivated) and a mutant version of apPol where E415 was mutated to alanine (apPol^E415A^) with either dGTP or GTP as the incoming nucleotide (Fig. 5E and Supplementary Fig. S6B and C). We found that, unlike apPol, apPol^E415A^ can incorporate both GTP and dGTP opposite dC (Fig. 5B), although GTP incorporation was slower than dGTP (Supplementary Fig. S6B and C). This indicates that E415 indeed plays a key role in discriminating GTP over dGTP as the incoming nucleotide. Recently it has been proposed that Q548 might act as the steric gate residue for apPol [23]. However, our primer extension assay shows that mutating Q548 to alanine did not enhance GTP incorporation, indicating that Q548 does not discriminate against GTP and (Fig. 5E and Supplementary Fig. S6C). Ternary3 is the only state where we could detect template overhang density for the T_+1_ position (Fig. 5B). N474 forms a hydrogen bond with the phosphate of T_+1_, thus making the base ordered. The T_+1_ base is in a flipped-out configuration, excluding it from the active site. This flipped-out configuration is essential to avoid a steric clash with Y481.

## Discussions

**Figure 6.**
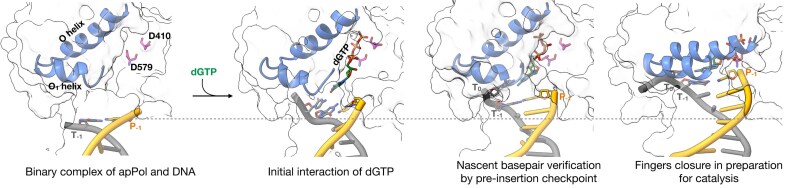
apPol backtracks during the pre-chemistry steps. Zoomed-in views of 3′ primer terminus in binary1, ternary1, ternary2, and ternary3 complexes (left to right) with the DNA primer (orange)/template (grey), O and O_1_ helices (blue) shown in ribbon representations. The dGTP (green), catalytic aspartates (magenta), and the P_−1_, T_−1_, and T_0_ bases are shown in stick representations. The rest of the apPol is shown as a translucent grey surface. The grey dashed line marks the position of P_−1_ base in binary1. All structures were aligned on the palm domain.

The series of cryoEM structures reported here (Fig. 6) captures how apPol transitions through the initial stages of interactions with its DNA and nucleotide substrates, ultimately forming a catalytically poised pre-chemistry ternary complex while maintaining Watson–Crick geometry of the nascent base pair. We discuss our results within the context of A-family polymerases and propose a mechanism by which apPol might accommodate DNA lesions within its active site while biassing the nascent base pair towards Watson–Crick geometry, a prerequisite for performing both replicative and lesion bypass synthesis within the same DNA polymerase.

### ApPol uses two fidelity checkpoints to accommodate TLS and replicative synthesis

**Figure 7.**
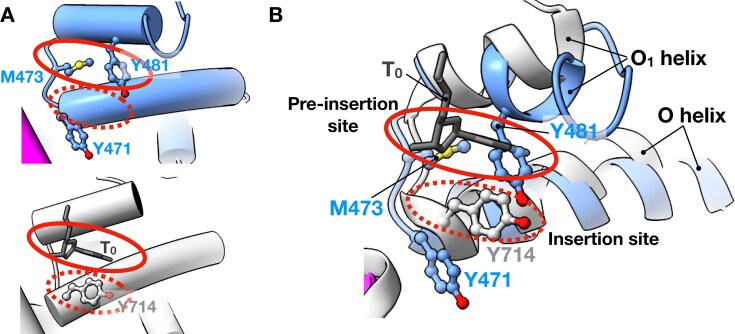
ApPol’s pre-insertion site is not accessible to the T_0_ base. (**A**) Close-up view of the pocket between the O and O_1_ helices of the fingers domain of the apPol binary1 complex (top) and BSt Pol I (bottom; PDB id: 1L3S). For clarity, the DNA is not shown, and the helices are shown as tubes. (**B**) Superposition of the pockets shown in panel (A) on the BSt Pol I binary structure (PDB id: 1L3S; white). ApPol colour coding is the same as Fig. 1B. BSt Pol I is shown in white and the T_0_ nucleotide of Pol I is shown in dark grey. The pre-insertion and insertion sites are highlighted with solid red and dashed red ovals, respectively. Y481, M473, and Y471 of apPol and Y714 of BSt Pol I are shown in ball-and-stick representation.

Pol I has been a model A-family replicative polymerase, and based on a series of structures of *B. stearothermophilus* Pol I (Bst Pol I) performing nucleotide addition *in crystallo*, a conformational coupling mechanism was proposed for high-fidelity replication [34, 55, 56]. In the pre-chemistry binary complex the O helix remains open with the insertion site (position of T_0_ base during catalysis) occupied by a tyrosine residue at the C-terminus of the O helix (Y714 in BSt Pol I) (Fig. 7). This tyrosine is conserved in all A-family polymerases and occupies the insertion site in most apo or binary A-family polymerase structures [49, 57–59], potentially preventing premature base pair formation with the fingers in the open configuration. In the fingers-open state of Bst Pol I, the T_0_ base remains lodged within a pocket formed by the O and O_1_ helix (pre-insertion site) (Fig. 7). As the polymerase moves from binary to ternary state, the O helix closes, leading to a conformational change in the loop connecting the O and O_1_ helices, which (a) blocks the pre-insertion site and (b) alters the orientation of Y714 and freeing the insertion site. Thus the T_0_ base is forced out of the pre-insertion site and into the newly available insertion site. Moreover, the altered conformation of the loop connecting the O and O_1_ helices forces the T_+1_ base to adopt a flipped-out orientation to avoid any steric clashes. These coupled conformational changes ensure that (a) no premature bond formation occurs with the fingers open, thus biasing the nascent base pair towards Watson–Crick geometry (since in the closed state the active site can accommodate predominantly Watson–Crick pairing), and (b) only a single templating base gets access to the insertion site during a single round of the catalysis, thereby reducing the chance of a frameshift error.

ApPol does not undergo the coupled conformational changes noted for Pol I. In the binary1 complex the pre-insertion site is blocked by M473 and Y481, excluding the T_0_ base from this position (Fig. 7 and Supplementary Fig. S3B). Moreover, the tyrosine at the C terminus of apPol’s O helix (Y471) does not occupy the insertion site (Fig. 7 and Supplementary Fig. S3B) and consequently apPol can form the nascent base pair with the fingers in the quasi-open state as seen in ternary1 and 2 (Fig. 4A and C; Movie 1). Since there are very few contacts between the templating base or the nascent base pair and apPol in binary1 and ternary1, respectively (Figs 2B and 4A), we anticipate nascent base pair formation with the fingers open to be independent of the identity of the bases. This might allow apPol to accommodate a nascent base pair that deviates from the Watson–Crick geometry, a feature that could aid in apPol’s TLS activity. Future structural work focusing on the conformational landscape of apPol during TLS will be essential for a detailed mechanistic understanding of apPol’s lesion bypass activities.

Base pairing in open configuration raises the question: how does apPol ensure fidelity during nucleotide incorporation? We hypothesize that apPol uses two fidelity checkpoints. The first checkpoint is captured by ternary2, where the nascent base pair fits within the snug pocket formed by Y481, Y485, and Y486 at the tip of the O_1_ helix (Fig. 4D and E and Supplementary Fig. S3E; Movie 4). This pocket is in fact a modified pre-insertion site. By modifying this site, apPol has converted the pre-insertion site into a pre-insertion checkpoint to assess the nascent base pair geometry. Consistent with this hypothesis, we found that mutating these three tyrosines to alanines reduced the accuracy of DNA synthesis (Fig. 4F). Moreover, using an assay measuring multiple nucleotide incorporation and strand displacement abilities of apPol, it has been reported that apPol^Y481A_Y485A_Y486A^ shows an overall reduced activity [22]. Notably, Y481, Y485, and Y486 are conserved in the apPol clade of the A-family but absent in other A-family polymerases studied to date (Supplementary Fig. S5) [22]. Another question is how might this pre-insertion checkpoint play a role in apPol’s TLS activities. Further structure–function correlation studies with the triple-mutant apPol will be essential to answer this question. The second checkpoint is captured by ternary3, where the nascent base pair is poised for catalysis and the fingers is in a closed conformation (Fig. 5A). In this state the active site will favour the Watson–Crick base pair geometry. These two fidelity checkpoints might allow apPol to perform TLS without sacrificing the accuracy of replicative synthesis.

### ApPol thumb is a master manipulator of DNA position

In all A-family polymerases studied to date, the DNA undergoes minimal movement between the binary and ternary complexes [34, 49, 59]. However, apPol is an exception to the rule with the enzyme backtracking with respect to the DNA [60] during transition from pre-chemistry binary to the catalytically poised pre-chemistry ternary state (Fig. 6; Movie 3). Going from binary1 to ternary1 the DNA moves towards the active site facilitated by a downward motion of the thumb (Fig. 3D; Movie 2), pushing the 3′ end of the primer towards the active site. Further, as apPol transitions from ternary1 to ternary2 and finally to ternary3, the DNA undergoes additional corkscrew motion, positioning the 3′ end of the primer into the active site (Fig. 3B and Supplementary Fig. S4; Movie 3).

This large-scale DNA movement is orchestrated in part by a rigid body motion of the thumb towards the fingers domain. In addition, the DNA slips with respect to the thumb to achieve the full extent of the movement. For instance, T319 of the thumb domain contacts the phosphate backbone of P_−3_ in binary1 and ternary1, P_−4_ in ternary2, and P_−5_ in ternary3 (Figs 2B, 4B, and 5B; Movie 6). As apPol progresses from binary1 to ternary3 the template strand forms contacts with residues from the palm and fingers and with the incoming nucleotide (Movie 5, Fig. 5A and B); alternating breakage and re-formation of the contacts with the thumb and residues in the vicinity of the active site leads to the DNA slippage.

Unlike other A-family polymerases, the thumb of apPol makes few contacts with the DNA, especially in the binary state, and this might explain the relatively weak affinity of DNA for apPol [5, 23]. For instance, Bst Pol I and T7 DNA polymerase binary complexes have polymerase–DNA interfaces of 1782 Å^2^ (PDB id: 1L3T) and 2009 Å^2^ (PDB id: 2AJQ), respectively, while the corresponding interface for apPol is only 607 Å^2^. For the apPol-specific pre-insertion checkpoint to function, the DNA and the incoming nucleotide need to undergo large-scale motions as apPol transitions from the pre-chemistry binary complex to the catalytically poised complex. The weaker contact between the apPol thumb and the DNA might have evolved to facilitate the large DNA motion enabling this apPol-specific checkpoint.

### Sugar pucker of the incoming dGTP

Recently, a pre-chemistry ternary complex structure of apPol with the fingers in closed configuration has been published (referred to as ternary4 from here onwards) [23]. The overall conformation of apPol is similar between ternary3 and ternary4, with the main difference being the orientation of the O helix. While the O helix of ternary4 is almost closed, the closure is not complete (Supplementary Fig. S6D). On the other hand, there are some critical differences in the orientation of the incoming dGTP. In ternary4, unlike other DNA polymerase structures that we are aware of, including ternary3, the ribose sugar of the incoming dGTP has been modelled with an O4′-endo sugar pucker (Supplementary Fig. S6A). The altered pucker in ternary4 compared to ternary3 alters the orientation of the incoming deoxyribose with respect to the 3′ end of the primer (Supplementary Fig. S6E) and results in several steric clashes, including a clash between C2′ of P_−1_ and C1′ of the incoming dGTP. It is puzzling why a matched incoming nucleotide (dGTP opposite dC in ternary4) would adopt an unusual sugar pucker. The published ternary4 structure was at an overall resolution of 3.2 Å. At this resolution it is challenging to unambiguously determine the sugar pucker and higher-resolution structures of ternary4 would be essential before any further analysis is possible.

### Why do the fingers close?

In most catalytically poised DNA polymerase ternary complexes, including apPol, the fingers domain stays in a closed conformation [45, 50, 61]. On the other hand, in the apo or binary states, the fingers are typically open. Closed fingers restrict the polymerization active site and sequester the 3′OH of the primer and incoming nucleotide from the bulk solvent, facilitating the phosphodiester bond formation. Nucleotide binding has typically been assigned as the trigger for fingers closure [62]. However, it has been shown that the Klenow fragment of *E. coli* Pol I and the herpes simplex virus-1 DNA polymerase can sample both the open and closed conformations in the absence of the incoming nucleotide [44, 58, 63]. This indicates that dNTP binding is not essential for fingers closing. It is possible that the closed state is stabilized in the presence of the correct incoming nucleotide and/or the two divalent metal ions associated with the nucleotide [44, 64, 65]. However, recent structures of the human leading strand DNA polymerase, polϵ, show that the pre-chemistry ternary complex with a correct nascent base pairing can exist with the fingers in open, closed, and intermediate states [66], indicating that even in the presence of the correct nucleotide, the fingers may stay open. These new observations raise the question: what triggers fingers closing? A combination of kinetic and time-resolved structural measurements investigating the short-lived pre-chemistry states of a DNA polymerase would be essential to answer this question, which is central to our understanding of the catalytic mechanisms of DNA polymerases.

### What happens after bond formation?

The series of structures presented here elucidate how apPol interacts with DNA and the incoming nucleotide to catalyse phosphodiester bond formation; however, we do not have direct structural insight into the post-chemistry events. While a post-chemistry binary complex (Supplementary Fig. S1A, Step 4) structure of apPol (generated through addition of a dideoxyATP to the 3′ end of the primer strand) has been reported [23], the modest resolution of 5.2 Å precludes drawing any atomistic inference. In fact, at this resolution, it is difficult to ascertain whether the structure is of a post-chemistry binary state or whether apPol has translocated (Supplementary Fig. S1A, Step 5) to a pre-chemistry binary form.

Nonetheless, based on the DNA position in binary1 and the published post-chemistry binary structure, we predict that, after chemistry apPol over-translocates, leading to the apPol–DNA pre-chemistry binary complex (binary1). Following this apPol backtracks to correct for the over-translocation and positions the templating DNA base in the active site.

Among the well-studied replication systems, the T7 bacteriophage replisome is perhaps the closest to the apicoplast replisome [67]. *In vitro* studies indicate that in T7 replisome the leading strand gp5 T7 DNA polymerase follows the gp4 helicase-primase closely with only a single nucleotide of the DNA template separating the two proteins [68, 69]. However, the over-translocation mechanism for apPol would require a larger slack in the DNA between apPol and the apicoplast helicase or any other partner protein positioned downstream of the polymerase. Alternatively, the over-translocation might be used by apPol only under special situations like lesion bypass, and during bulk DNA synthesis, a close contact with a partner protein like the helicase might prevent apPol from over-translocating. Understanding the molecular mechanism of apPol’s post-chemistry substrate reorientation and its implications for the apicoplast replisome organization would be vital for developing a clear picture of how the apicoplast genome is copied.

## Supplementary Material

gkaf1005_Supplemental_Files

## Data Availability

The following cryoEM maps and atomic coordinates have been deposited to EMData Bank (EMDB) and Protein Data Bank (PDB), respectively. Consensus refinement (EMD-53335 and 9QSC), binary1 (EMD-53378 and 9QUJ), binary2 (EMD-53379 and 9QUN), ternary1 (EMD-53376 and 9QUA), ternary2 (EMD-53374 and 9QU8), and ternary3 (EMD-53391 and 9QV9).
